# ﻿A checklist of helminths (Acanthocephala, Nematoda and Platyhelminthes) found in rodents (Mammalia, Rodentia) from Slovakia

**DOI:** 10.3897/zookeys.1251.154911

**Published:** 2025-09-04

**Authors:** Yaroslav Syrota, Zuzana Hurníková, Vitaliy Kharchenko, Martina Miterpáková

**Affiliations:** 1 Institute of Parasitology, Slovak Academy of Sciences, Hlinkova 3, 040 01 Košice, Slovakia Institute of Parasitology, Slovak Academy of Sciences Košice Slovakia; 2 I. I. Schmalhausen Institute of Zoology, National Academy of Sciences of Ukraine, vul. B. Khmelnytskoho, 15, 01054 Kyiv, Ukraine I. I. Schmalhausen Institute of Zoology, National Academy of Sciences of Ukraine Kyiv Ukraine

**Keywords:** Biodiversity, Europe, inventory, micromammal, parasites, review, Slovak Republic

## Abstract

Nearly seven decades have passed since the first records of helminths in rodents in Slovakia were published. Thereafter, the number of documented helminth species within the territory of the country has steadily increased. This checklist compiles data from 34 sources, including 27 academic articles, five unpublished reports, and two conference proceedings produced between 1955 and early 2025. Following a critical review of each source, 73 nominal helminth species from 27 species of rodents, comprising 30 nematodes, 29 cestodes, 13 trematodes, and one acanthocephalan, were included in this checklist. Information on helminth diversity is absent for only two of the 29 rodent species registered in the Slovak Republic: *Myocastor coypus* and *Arvicola scherman*. Furthermore, our findings suggest that certain rodent species remain under-researched despite the presence of some information on their helminth fauna. This checklist lays the basis for future helminthological research on rodents in Slovakia. The raw dataset generated for this article is available in the Suppl. material 1.

## ﻿Introduction

Rodents are the most diverse order of Mammalia, with over 2000 species inhabiting terrestrial, subterranean, arboreal, and aquatic environments on all continents except Antarctica (Wolff and Sherman 2007). Recent research estimates that the biomass of all land mammals is 22 million tonnes (wet weight), with rodents comprising 16% of this total (Greenspoon et al. 2023). Due to their wide distribution and significant biomass, rodents play a vital role in the trophic networks in many terrestrial ecosystems. In addition to being prey for various predators, they also host many species of helminths, a subject of considerable research interest, as evidenced by publications summarising numerous studies on helminths of rodents (Smales 1997; Preisser and Falcón-Ordaz 2019; Preisser 2019; Islam et al. 2020; Panti-May et al. 2021; Dursahinhan et al. 2023; Hamzavi et al. 2024).

In Slovakia, as in other countries, studies have also been conducted on the diversity of rodent helminths. To our knowledge, the first summarising work on this topic is a publication indexing a museum collection of helminths from hosts of different taxa, including data on rodent helminths’ findings from the territory of the former Czechoslovakia (Hovorka 1968). Another valuable resource is several papers within the Synopsis of Cestodes in Slovakia series (Macko et al. 1994; Hanzelová et al. 1995; Hanzelová and Ryšavý 1996). These articles, which cover all groups of vertebrate hosts, also include records of rodent cestodes and their hosts. The most recent work providing data on parasites is a comprehensive review of mammals in Slovakia (Krištofík and Danko 2012). Although the monograph offers a list of endo- and ectoparasites found in each mammal species recorded in Slovakia, its primary focus is on the mammals rather than their parasites.

Consequently, the available literature includes five sources with limited inventories on rodent helminth diversity in the country, and none are exclusively dedicated to the helminths of these hosts. Our study aims to fill this gap by compiling up-to-date information on helminths of wild and synanthropic rodents inhabiting Slovakia.

## ﻿Material and methods

The checklist was compiled based on information obtained from two sources: (1) an online search of academic research databases such as Scopus and Web of Science, and (2) a bibliographical search of literature in the library of the Institute of Parasitology of the Slovak Academy of Sciences. All found sources were critically reviewed before their data were included in this checklist. One subject of this review was eliminating geographical ambiguity, as in several instances, it was unclear whether the data originated from Czechia or Slovakia. This uncertainty arose because part of the research had been conducted in former Czechoslovakia without indicating a precise location. Such sources were excluded. Another criterion for selecting a source was the originality of the data. In some cases, an author had published nearly identical content in two sources: an article and a conference abstract. Only sources with a more comprehensive dataset were selected for such cases. As a result of the selection, 34 sources produced between 1955 and the beginning of 2025 were used to compile the checklist; 27 of these sources are published academic works, five are unpublished reports from the Institute of Parasitology, and two are abstracts from conference proceedings. The raw dataset used to create the checklist is available as the Suppl. material 1.

**Figure 1.**
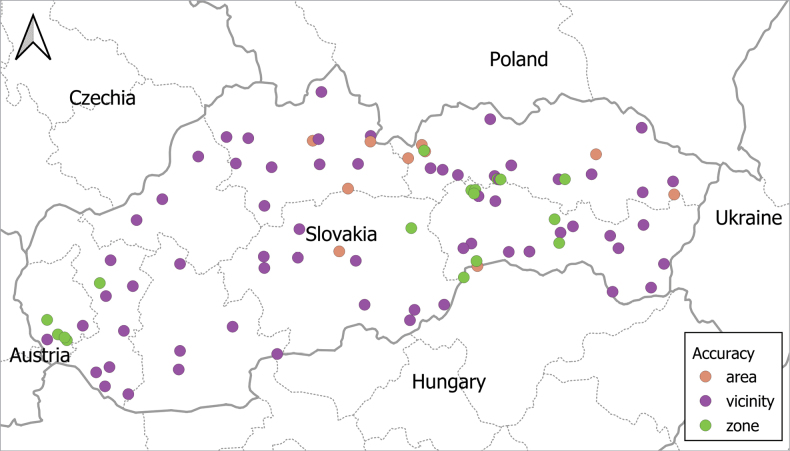
Approximate geographic locations in Slovakia where rodents were sampled. Note: Sampling localities are categorised based on the precision of their geographic coordinates: zone – precise locations, vicinity – areas near landmarks, area – broad or vaguely defined locations.

The studies used for this checklist varied considerably in the precision with which localities were described, from specific sites to large regions; however, most sources provided no exact geographic coordinates. To standardise the presentation of geographical information, we used the Google Maps web application to determine approximate coordinates for each locality. We used approximate centres for larger areas, such as administrative regions or conservation zones. Localities were categorised into three groups based on precision: zone – precise locations; vicinity – areas near landmarks; and area – broad or vaguely defined locations. Detailed information for each locality, including name, coordinates, and precision category, is available in the Suppl. material 1. Coordinates provided by us should be regarded as the best available cartographic proxies for the named sites rather than instrument-logged GPS points. We used QGIS® software to visualise the geographical coverage of helminth research across Slovakia (Fig. 1).

The parasite-host list is presented alphabetically, starting with the acanthocephalans, followed by the nematodes and the platyhelminths (cestodes and trematodes). The list includes information on synonyms of helminth scientific names, hosts (listed in alphabetical order), localities (listed in alphabetical order), infection sites (listed in alphabetical order), references, and remarks (if necessary). Scientific names of helminths follow those provided in the Global Biodiversity Information Facility (GBIF.org 2024), Global Cestode Database (Caira et al. 2022), World Register of Marine Species (WoRMS Editorial Board 2024), Nemys: World Database of Nematodes (Nemys 2024), and selected publications (Biserkov 1998; Mahesh Kumar et al. 2006; Jackson and Friberg 2022; Makarikov and Binkienė 2022; Behnke and Jackson 2024). A ‘Synonyms’ entry is provided only when the original binomen in a source differs from the combination accepted in the databases and literature used for name verification.

In addition, a host-parasite list was compiled based on the parasite-host list. This list details each host, including its scientific name and the scientific names of all associated helminths, organised by taxonomic group: Acanthocephala, Nematoda, Cestoda, and Trematoda. Within each taxonomic group, the helminth names are listed in alphabetical order.

## ﻿Results

Based on the literature analysis, we documented 73 helminth species from 27 rodent host species in Slovakia. Additionally, we considered each of the genera *Echinostoma*, *Mesocestoides*, *Citellina*, and *Toxocara* to be represented by at least one species, as no species-level identifications were available for these taxa in the checklist. As a result, at least four additional species can be inferred, bringing the total number of detected species to a minimum of 77. These include 32 species of nematodes, 30 species of cestodes, 14 species of trematodes, and one species of acanthocephalans.

### ﻿Annotated parasite-host list

#### ﻿Phylum Acanthocephala Rudolphi, 1802

**Class Archiacanthocephala Meyer, 1931**

**Order Moniliformida Schmidt, 1972**

**Family Moniliformidae Van Cleave, 1924**

**Genus *Moniliformis* Travassos, 1915**

##### *Moniliformis moniliformis* (Bremser, 1811)

**Hosts.***Apodemus flavicollis*, *Apodemus sylvaticus*, *Glis glis*, *Microtus arvalis*, *Mus musculus*, *Myodes glareolus*, *Rattus norvegicus*, *Spermophilus citellus*.

**Localities.** Domica, Jablonov, Košice, Protected Landscape Area (PLA) Poľana, Rožňava, Slovenský kras, Vihorlat, Vysoké Tatry, Žiar nad Hronom.

**Infection site.** Small intestine.

**Reported by.**Erhardová (1956, 1958), Baruš and Tenora (1957), Tenora (1963), Mituch (1970, 1975), Mituch et al. (1987).

#### ﻿Phylum Nematoda Cobb, 1932

**Class Chromadorea Inglis, 1983**

**Order Rhabditida Chitwood, 1933**

**Family Ascarididae Baird, 1853**

**Genus *Porrocaecum* Railliet & Henry, 1912**

##### *Porrocaecum depressum* (Zeder, 1800)

**Hosts.***Apodemus sylvaticus*, *Mus musculus*, *Sciurus vulgaris*.

**Localities.** PLA Poľana, Roháčska dolina.

**Infection site.** Body cavity, caecum, large intestine.

**Reported by.**Tenora (1967), Mituch (1975).

**Remark.***P. depressum* has a cosmopolitan distribution and has been reported from approximately 47 species of birds of prey (Atkinson et al. 2008). Rodents serve as paratenic hosts.

##### *Porrocaecum* spp.

**Hosts.***Microtus agrestis*, *Microtus subterraneus*.

**Localities.** Roháčska dolina, Vysoké Tatry.

**Infection site.** Caecum, small intestine wall – nodules.

**Reported by.**Erhardová (1956), Tenora (1967).

**Remark.** Members of the genus *Porrocaecum* are widely distributed intestinal parasites of birds, with shrews – and perhaps other small mammals that consume intermediate hosts (e.g., earthworms) – serving as paratenic hosts to transfer the parasite to carnivorous definitive hosts (Anderson 2000).

#### ﻿Genus *Toxocara* Stiles & Hassall, 1905

##### *Toxocara* spp.

**Hosts.***Apodemus agrarius*, *Apodemus flavicollis*, *Apodemus sylvaticus*, *Apodemus uralensis*, *Micromys minutus*, *Microtus arvalis*, *Mus musculus*, *Myodes glareolus*.

**Localities.** Košice, Tatra National Park (TANAP).

**Infection site.** Antibodies in sera.

**Reported by.**Dubinský et al. (1995).

#### ﻿Family Heligmosomidae Cram, 1927

**Genus *Carolinensis* Travassos, 1937**

##### *Carolinensis minutus* (Dujardin, 1845)

**Hosts.***Microtus arvalis*, *Microtus subterraneus*, *Myodes glareolus*.

**Locality.** Čergov Mts.

**Infection site.** Not specified.

**Reported by.**Mészáros and Štolmann (1984).

#### ﻿Genus *Glistrongylus* Durette Desset, Digiani, Kilani & Geffard Kuriyama, 2017

##### *Glistrongylus gracilis* (Leuckart, 1842)

**Synonym.***Longistriata schulzi* Schachnasarova, 1949.

**Hosts.***Apodemus sylvaticus*, *Dryomys nitedula*, *Glis glis*, *Microtus arvalis*.

**Localities.** PLA Poľana, Roháčska dolina, Slovenský kras, Vysoké Tatry.

**Infection site.** Small intestine.

**Reported by.**Baruš and Tenora (1957), Tenora (1967), Mituch (1970, 1975).

#### ﻿Genus *Heligmosomoides* Hall, 1916

##### *Heligmosomoides glareoli* Baylis, 1928

**Synonym.***Heligmosomum tatricum* Erchardova, 1955.

**Hosts.***Apodemus flavicollis*, *Apodemus sylvaticus*, *Chionomys nivalis*, *Microtus agrestis*, *Microtus subterraneus*, *Microtus tatricus*, *Myodes glareolus*.

**Localities.** Čergov Mts., PLA Poľana, Roháčska dolina, Vysoké Tatry.

**Infection site.** Small intestine.

**Reported by.**Erhardová (1955a), Tenora (1955, 1967), Mituch (1970, 1975), Mészáros and Štolmann (1984), Mituch et al. (1987).

##### *Heligmosomoides polygyrus* (Dujardin, 1845)

**Synonym.***Heligmosomum skrjabini* Skrjabin & Schikhobalova, 1952.

**Hosts.***Apodemus agrarius*, *Apodemus flavicollis*, *Apodemus sylvaticus*, *Apodemus uralensis*, *Microtus arvalis*, *Microtus oeconomus*, *Microtus subterraneus*, *Mus musculus*, *Mus spicilegus*, *Muscardinus avellanarius*, *Myodes glareo­lus*, *Sicista betulina*.

**Localities.** Bratislava, Čergov Mts., Domica, Gabčíkovo, Košice, Liptovský Mikuláš, Orava, PLA Poľana, Roháčska dolina, Rozhanovce, South Eastern Slovakia, South Slovakia, Veľké Kapušany, Vysoké Tatry, Zádiel, Žiar nad Hronom.

**Infection site.** Intestines, small intestine, small intestine – muscularis propria.

**Reported by.**Erhardová (1955a, 1955b, 1956), Tenora (1963, 1967), Mituch (1970, 1975), Mészáros and Štolmann (1984), Mituch et al. (1987), Ribas et al. (2009), Ondríková et al. (2010).

#### ﻿Genus *Heligmosomum* Railliet & Henry, 1909

##### *Heligmosomum costellatum* (Dujardin, 1845)

**Hosts.***Apodemus flavicollis*, *Apodemus sylvaticus*, *Apodemus uralensis*, *Chionomys nivalis*, *Microtus agrestis*, *Microtus arvalis*, *Microtus subterraneus*, *Microtus tatricus*, *Mus musculus*, *Myodes glareolus*.

**Localities.** Bratislava, Čergov Mts., Domica, Kráľovský Chlmec, PLA Poľana, Roháčska dolina, Vysoké Tatry.

**Infection site.** Small intestine, stomach.

**Reported by.**Erhardová (1955a, 1956), Tenora (1955, 1963, 1967), Mituch (1970, 1975), Mészáros and Štolmann (1984), Mituch et al. (1987).

##### *Heligmosomum halli* (Schulz, 1926)

**Hosts.***Microtus agrestis*, *Microtus arvalis*, *Microtus subterraneus*, *Microtus tatricus*, *Myodes glareolus*.

**Localities.** PLA Poľana, Vysoké Tatry.

**Infection site.** Intestines, small intestine.

**Reported by.**Mituch (1970, 1975).

##### *Heligmosomum mixtum* Schulz, 1929

**Host.***Myodes glareolus*.

**Locality.** Čergov Mts.

**Infection site.** Small intestine.

**Reported by.**Mészáros and Štolmann (1984).

#### ﻿Family Heterakidae Railliet & Henry, 1912

**Genus *Heterakis* Schrank, 1790**

##### *Heterakis spumosa* Schneider, 1866

**Synonym.***Ganguloterakis spumosa* (Schneider, 1866).

**Hosts.***Apodemus agrarius*, *Rattus norvegicus*.

**Localities.** Banská Bystrica, Bratislava, Bytča, Galanta, Levice, Púchov, Rajec, Rozhanovce, Sečovce, Senec, Trenčín, Trnava, Turčianske Teplice.

**Infection site.** Caecum, large intestine, small intestine.

**Reported by.**Mituch (1957), Erhardová (1958), Ondríková et al. (2010).

#### ﻿Family Heteroxynematidae Skrjabin & Shikhobalova, 1948

**Genus *Aspiculuris* Schulz, 1927**

##### *Aspiculuris dinniki* Schulz, 1927

**Hosts.***Apodemus flavicollis*, *Apodemus sylvaticus*, *Apodemus uralensis*, *Chionomys nivalis*, *Microtus agrestis*, *Microtus subterraneus*, *Microtus tatricus*, *Myodes glareolus*.

**Localities.** PLA Poľana, Roháčska dolina, Vysoké Tatry.

**Infection site.** Caecum, large intestine, small intestine.

**Reported by.**Erhardová (1955a), Tenora (1955, 1967), Mituch (1970, 1975), Mituch et al. (1987).

##### *Aspiculuris tetraptera* Nitzsch, 1821

**Hosts.***Apodemus flavicollis*, *Apodemus sylvaticus*, *Mus musculus*.

**Localities.** PLA Poľana, Vysoké Tatry.

**Infection site.** Caecum, large intestine.

**Reported by.**Erhardová (1955a), Mituch (1970, 1975), Mituch et al. (1987).

#### ﻿Family Oxyuridae Weinland, 1858

**Genus *Citellina* Prendel, 1928**

##### *Citellina* spp.

**Host.***Spermophilus citellus*.

**Localities.** Boleráz – airport, Bratislava – Zelená voda, Spišský hrad – castle.

**Infection site.** Intestines.

**Reported by.**Štrkolcová et al. (2024).

#### ﻿Genus *Enterobius* Leach, 1853

##### Enterobius (Enterobius) sciuri (Cameron, 1932)

**Host.***Sciurus vulgaris*.

**Localities.** PLA Poľana, Vysoké Tatry.

**Infection site.** Caecum, large intestine.

**Reported by.**Mituch (1970, 1975), Mituch et al. (1987).

#### ﻿Genus *Syphacia* Seurat, 1916

##### Syphacia (Seuratoxyuris) frederici (Roman, 1945)

**Host.***Apodemus flavicollis*.

**Locality.** Čergov Mts.

**Infection site.** Not specified.

**Reported by.**Mészáros and Štolmann (1984).

##### Syphacia (Syphacia) montana (Yamaguti, 1943)

**Hosts.***Marmota marmota*, *Microtus agrestis*, *Microtus subterraneus*, *Microtus tatricus*, *Myodes glareolus*.

**Localities.** Čergov Mts., Roháčska dolina, Vysoké Tatry.

**Infection site.** Caecum, small intestine.

**Reported by.**Tenora (1967), Mituch (1970), Mészáros and Štolmann (1984), Mituch et al. (1987).

##### Syphacia (Syphacia) nigeriana (Baylis, 1928)

**Host.***Microtus arvalis*.

**Locality.** Čergov Mts.

**Infection site.** Not specified.

**Reported by.**Mészáros and Štolmann (1984).

##### Syphacia (Syphacia) obvelata (Rudolphi, 1802)

**Hosts.***Apodemus agrarius*, *Apodemus flavicollis*, *Apodemus sylvaticus*, *Chionomys nivalis*, *Microtus agrestis*, *Microtus arvalis*, *Microtus oeconomus*, *Microtus subterraneus*, *Mus musculus*, *Mus spicilegus*, *Muscardinus avellanarius*, *Myodes glareolus*.

**Localities.** Domica, Gabčíkovo, Košice, Kráľovský Chlmec, Liptovský Mikuláš, Medzilaborce, Michalovce, Nízke Tatry, Orava, PLA Poľana, Prešov, Rožňava, Slovenské Nové Mesto, South Eastern Slovakia, Vysoké Tatry, Žiar nad Hronom.

**Infection site.** Caecum, intestines, large intestine, small intestine.

**Reported by.**Erhardová (1955a, 1956, 1958), Tenora (1955, 1963), Mituch (1970, 1975), Mituch et al. (1987), Ribas et al. (2009).

**Remark.** Genetic evidence indicates that *S. obvelata* is associated with murine rodents (Okamoto et al. 2007; Stewart et al. 2018); therefore, reports of its occurrence in other rodent groups are doubtful and should likely be considered misidentifications or incidental infections.

##### Syphacia (Syphacia) stroma (Linstow, 1884)

**Hosts.***Apodemus agrarius*, *Apodemus flavicollis*.

**Locality.** Rozhanovce.

**Infection site.** Not specified.

**Reported by.**Ondríková et al. (2010).

#### ﻿Family Rhabditidae

**Genus *Pelodera* Schneider, 1866**

##### *Pelodera orbitalis* (Sudhaus & Schulte, 1986)

**Synonym.***Rhabditis orbitalis* Sudhaus & Schulte, 1986.

**Hosts.***Chionomys nivalis*, *Microtus agrestis*, *Microtus arvalis*, *Microtus subterraneus*, *Microtus tatricus*, *Myodes glareolus*.

**Localities.** Belianske Tatry, Nízke Tatry, Roháče, Slovenský kras, Vysoké Tatry.

**Infection site.** Conjunctival sacs, orbit.

**Reported by.**Baruš and Hrabě (1991), Kocianová-Adamcová et al. (2006).

#### ﻿Family Rictulariidae Railliet, 1916

**Genus *Rictularia* Froelich, 1802**

##### *Rictularia proni* Seurat, 1915

**Hosts.***Apodemus flavicollis*, *Apodemus sylvaticus*, *Dryomys nitedula*, *Eliomys quercinus*, *Microtus arvalis*, *Mus musculus*, *Myodes glareolus*.

**Localities.** Bratislava, Jablonov, Roháčska dolina, Slovenský kras, Vysoké Tatry.

**Infection site.** Small intestine, stomach.

**Reported by.**Baruš and Tenora (1957), Erhardová (1958), Tenora (1967), Mituch (1970).

##### *Rictularia* spp.

**Host.***Myodes glareolus*.

**Locality.** Roháčska dolina.

**Infection site.** Small intestine.

**Reported by.**Tenora (1967).

#### ﻿Family Spirocercidae Chitwood & Wehr, 1932

**Genus *Mastophorus* Diesing, 1853**

##### *Mastophorus muris* (Gmelin, 1790)

**Hosts.***Apodemus agrarius*, *Chionomys nivalis*, *Microtus subterraneus*, *Mus musculus*, *Myodes glareolus*, *Rattus norvegicus*.

**Localities.** PLA Poľana, Roháčska dolina, Rozhanovce, Vysoké Tatry.

**Infection site.** Stomach.

**Reported by.**Tenora (1967), Mituch (1970, 1975), Mituch et al. (1987), Ondríková et al. (2010).

#### ﻿Family Strongyloididae Chitwood & McIntosh, 1934

**Genus *Strongyloides* Grassi, 1879**

##### *Strongyloides ratti* Sandground, 1925

**Host.***Rattus norvegicus*.

**Localities.** Dunajská Streda, Galanta, Košice, Kráľovský Chlmec, Levice, Nové Zámky, Šahy, Šurany.

**Infection site.** Small intestine.

**Reported by.**Mituch (1957).

#### ﻿Family Trichostrongylidae Leiper, 1912

**Genus *Travassosius* Khalil, 1922**

##### *Travassosius rufus* Khalil, 1922

**Host.***Castor fiber*.

**Localities.** Danube River, Laborec River, Topľa River.

**Infection site.** Intestines.

**Reported by.**Bystrianska et al. (2021).

#### ﻿Genus *Trichostrongylus* Looss, 1905

##### *Trichostrongylus retortaeformis* (Zeder, 1800)

**Hosts.***Microtus arvalis*, *Sciurus vulgaris*.

**Locality.** Vysoké Tatry.

**Infection site.** Intestines.

**Reported by.**Mituch (1970).

**Remark.** Lagomorphs are the primary hosts of *T. retortaeformis*, as shown by Audebert et al. (2002); rodent records likely represent accidental spill-over infections rather than stable host–parasite associations.

##### Trichostrongylidae gen. sp.

**Host.***Marmota marmota*, *Spermophilus citellus*.

**Localities.** Bratislava – Štefánikova mohyla, Bratislava – Zelená voda, Chtelnica, Detvianska Huta, Gemerské Dechtáre, Jesenské, Košice – airport, Kružná, Moldava – hill, Moldava nad Bodvou, Petrovce – airport, Pieskovec – east, Pieskovec zásoby, Poráč, Silica pod Búčnikom, Spišská Nová Ves – Hrádok, Velická dolina.

**Infection site.** Intestines.

**Reported by.**Jászayová et al. (2023), Štrkolcová et al. (2024).

#### ﻿Class Enoplea Inglis, 1983

**Order Trichinellida Hall, 1916**

**Family Capillariidae Railliet, 1915**

**Genus *Aonchotheca* López-Neyra, 1947**

##### *Aonchotheca annulosa* (Dujardin, 1845)

**Synonyms.***Capillaria annulosa* (Dujardin, 1845), *Capillaria murisylvatici* (Diesing, 1851).

**Hosts.***Apodemus flavicollis*, *Apodemus sylvaticus*, *Microtus arvalis*, *Mus musculus*, *Myodes glareolus*, *Rattus norvegicus*.

**Localities.** Čergov Mts., Gabčíkovo, Kráľovský Chlmec, Nové Mesto nad Váhom, PLA Poľana, Rajec, Rozhanovce, Sečovce, Vihorlat, Vysoké Tatry.

**Infection site.** Intestines, small intestine, stomach.

**Reported by.**Erhardová (1956), Mituch (1957, 1970, 1975), Mészáros and Štolmann (1984), Mituch et al. (1987), Ondríková et al. (2010).

##### *Aonchotheca myoxinitelae* (Diesing, 1851)

**Synonym.***Skrjabinocapillaria myoxi-nitelae* (Diesing, 1851).

**Host.***Eliomys quercinus*.

**Locality.** Slovenský kras.

**Infection site.** Not specified.

**Reported by.**Baruš and Tenora (1957).

#### ﻿Genus *Calodium* Dujardin, 1845

##### *Calodium hepaticum* (Bancroft, 1893)

**Synonyms.***Capillaria hepatica* Bancroft, 1893, *Hepaticola hepatica* (Bancroft, 1893).

**Hosts.***Apodemus flavicollis*, *Apodemus sylvaticus*, *Arvicola amphibius*, *Chionomys nivalis*, *Microtus agrestis*, *Microtus arvalis*, *Myodes glareolus*, *Rattus norvegicus*.

**Localities.** Košice – zoo, Lučenec, PLA Poľana, Sečovce, TANAP, Trenčín, Vihorlat, Vysoké Tatry.

**Infection site.** Liver.

**Reported by.**Erhardová (1956), Mituch (1957, 1970, 1975), Mituch et al. (1987), Miterpáková et al. (2024).

#### ﻿Genus *Capillaria*

##### *Capillaria* spp.

**Host.***Spermophilus citellus*.

**Localities.** Bratislava – airport, Chtelnica, Ďurkovec – camp, Ďurkovec – skilift, Jesenské, Košice – airport, Kružná, Kuchyňa vývrat, Moldava – hill, Moldava nad Bodvou habitation, Muráň Biele Vody, Petrovce – airport, Pieskovec – east, Pieskovec zásoby, Poráč, Silica pod Búčnikom, Spišská Kapitula, Spišská Nová Ves – airport, Spišská Nová Ves – Hrádok, Spišské Podhradie, Spišský hrad – castle, Teplička, Vyšný Slavkov.

**Infection site.** Intestines.

**Reported by.**Štrkolcová et al. (2024).

#### ﻿Genus *Liniscus* Dujardin, 1845

##### *Liniscus papillosus* (Polonio, 1860)

**Synonym.***Capillaria papillosa* (Polonio, 1860).

**Host.***Rattus norvegicus*.

**Localities.** Bytča, Kráľovský Chlmec, Ružomberok, Trebišov.

**Infection site.** Urinary bladder.

**Reported by.**Mituch (1957).

#### ﻿Family Trichinellidae Ward, 1907

**Genus *Trichinella* Railliet, 1895**

##### *Trichinella britovi* Pozio, La-Rosa, Murrell & Lichtenfels, 1992

**Host.***Myodes glareolus*.

**Locality.** Vysoké Tatry.

**Infection site.** Muscles.

**Reported by.** Hurníková et al. (2025)

##### *Trichinella pseudospiralis* Garkavi, 1972

**Hosts.***Apodemus flavicollis*, *Rattus norvegicus*.

**Localities.** Michalovce, Vysoké Tatry.

**Infection site.** Muscles.

**Reported by.**Hurníková et al. (2005, 2025).

##### *Trichinella* spp.

**Hosts.***Apodemus flavicollis*, *Rattus norvegicus*.

**Localities.** Humenné, Košice, Prešov, Stará Ľubovňa.

**Infection site.** Muscles.

**Reported by.**Mituch (1956, 1957).

#### ﻿Family Trichosomoididae Yorke & Maplestone, 1926

**Genus *Trichosomoides* Railliet, 1895**

##### *Trichosomoides crassicauda* (Bellingham, 1840)

**Host.***Rattus norvegicus*.

**Localities.** Bratislava, Galanta, Hlohovec.

**Infection site.** Urinary bladder.

**Reported by.**Mituch (1957), Erhardová (1958).

#### ﻿Family Trichuridae Railliet, 1915

**Genus *Trichuris* Roederer, 1761**

##### *Trichuris muris* (Schrank, 1788)

**Hosts.***Apodemus flavicollis*, *Apodemus sylvaticus*, *Microtus agrestis*, *Microtus arvalis*, *Microtus oeconomus*, *Mus musculus*, *Rattus norvegicus*.

**Localities.** Bratislava, Domica, Gabčíkovo, Košice, Kráľovský Chlmec, Levice, PLA Poľana, Rožňava, Ružomberok, Sečovce, Trebišov, Vysoké Tatry.

**Infection sites.** Caecum, intestines, large intestine.

**Reported by.**Erhardová (1955a, 1956, 1958), Mituch (1957, 1970, 1975), Tenora (1963), Mituch et al. (1987).

**Remark.***Trichuris arvicolae*Feliu et al., 2000 was described from arvicoline rodents, whereas *T. muris* is suggested to be specialised to murine rodents (Feliu et al. 2000). Accordingly, the status of the specimens from arvicoline rodents should be re-evaluated since they probably belong to *T. arvicolae*.

#### ﻿Phylum Platyhelminthes Minot, 1876

**Class Cestoda van Beneden, 1849**

**Order Cyclophyllidea van Beneden in Braun, 1900**

**Family Anoplocephalidae Cholodkovsky, 1902**

**Genus *Anoplocephaloides* Baer, 1923**

##### *Anoplocephaloides dentata* (Galli-Valerio, 1905)

**Synonyms.***Paranoplocephala dentata*Galli-Valerio, 1905, *Paranoplocephala brevis* Kirschenblatt, 1938.

**Hosts.***Apodemus flavicollis*, *Chionomys nivalis*, *Microtus agrestis*, *Microtus arvalis*, *Microtus subterraneus*, *Microtus tatricus*, *Myodes glareolus*.

**Localities.** Čergov Mts., Domica, Nízke Tatry, PLA Poľana, Roháče, Roháčska dolina, Vysoké Tatry.

**Infection sites.** Caecum, intestines, small intestine.

**Reported by.**Tenora (1955, 1967), Erhardová (1955a, 1956, 1958), Mituch (1970, 1975), Tenora and Murai (1980), Mészáros and Štolmann (1984), Mituch et al. (1987).

#### ﻿Genus *Ctenotaenia* Railliet, 1893

##### *Ctenotaenia marmotae* (Frölich, 1802)

**Host.***Marmota marmota*.

**Locality.** Velická dolina, Vysoké Tatry.

**Infection site.** Small intestine.

**Reported by.**Tenora (1961), Jászayová et al. (2023).

#### ﻿Genus *Eurotaenia* Haukisalmi, Hardman, Hoberg & Henttonen, 2014

##### *Eurotaenia gracilis* (Tenora & Murai, 1980)

**Synonym.***Paranoplocephala gracilis* Tenora & Murai, 1980.

**Hosts.***Chionomys nivalis*, *Microtus agrestis*, *Microtus subterraneus*, *Microtus tatricus*, *Myodes glareolus*.

**Localities.** Mutné, Roháče.

**Infection site.** Small intestine.

**Reported by.**Tenora and Murai (1980).

#### ﻿Genus *Microticola* Haukisalmi, Hardman, Hoberg & Henttonen, 2014

##### *Microticola blanchardi* (Moniez, 1891)

**Synonym.***Paranoplocephala blanchardi* (Moniez, 1891).

**Hosts.***Microtus subterraneus*, *Microtus tatricus*, *Myodes glareolus*.

**Locality.** Roháče.

**Infection site.** Small intestine.

**Reported by.**Tenora and Murai (1980).

#### ﻿Genus *Paranoplocephala* Lühe, 1910

##### *Paranoplocephala macrocephala* (Douthitt, 1915)

**Synonym.***Andrya macrocephala* Douthitt, 1915.

**Hosts.***Apodemus flavicollis*, *Apodemus sylvaticus*, *Arvicola amphibius*, *Chionomys nivalis*, *Microtus agrestis*, *Microtus arvalis*, *Microtus oeconomus*, *Microtus subterraneus*, *Microtus tatricus*, *Mus musculus*, *Myodes glareolus*.

**Localities.** Gabčíkovo, PLA Poľana, Roháčska dolina, Veľký Meder, Vysoké Tatry.

**Infection site.** Small intestine.

**Reported by.**Erhardová (1956, 1958), Tenora (1963, 1967), Mituch (1970, 1975).

**Remark.** According to Haukisalmi and Henttonen (2003), *P. macrocephala* is a parasite of microtine and geomyid rodents in North America. The taxonomic position of cestodes from Europe identified as *P. macrocephala* requires further studies.

##### *Paranoplocephala microti* (Hansen, 1947)

**Synonym.***Andrya microti* Hansen, 1947.

**Hosts.***Microtus arvalis*, *Myodes glareolus*.

**Localities.** Domica, Košice.

**Infection site.** Small intestine.

**Reported by.**Erhardová (1956).

**Remark.** According to Haukisalmi et al. (2014), *P. microti* is a parasite of voles in North America only. The taxonomic position of cestodes from Slovakia identified as *P. microti* needs to be confirmed by further examinations.

##### *Paranoplocephala omphalodes* (Hermann, 1783)

**Hosts.***Apodemus agrarius*, *Apodemus flavicollis*, *Apodemus sylvaticus*, *Chionomys nivalis*, *Microtus agrestis*, *Microtus arvalis*, *Microtus subterraneus*, *Microtus tatricus*, *Myodes glareolus*, *Rattus norvegicus*.

**Localities.** Bratislava, Domica, Eastern Slovakia, Orava, PLA Poľana, Roháče, Vysoké Tatry.

**Infection site.** Intestines, large intestine, small intestine.

**Reported by.**Erhardová (1955a, 1956, 1958), Tenora (1955), Mituch (1970, 1975), Tenora and Murai (1980), Mituch et al. (1987).

##### *Paranoplocephala* spp.

**Hosts.***Marmota marmota*, *Microtus subterraneus*, *Myodes glareolus*.

**Localities.** Čergov Mts., Vysoké Tatry.

**Infection site.** Small intestine.

**Reported by.**Mészáros and Štolmann (1984), Mituch et al. (1987).

#### ﻿Family Catenotaeniidae Spasskii, 1950

**Genus *Catenotaenia* Janicki, 1904**

##### *Catenotaenia dendritica* (Goeze, 1782)

**Host.***Sciurus vulgaris*.

**Localities.** PLA Poľana, Vysoké Tatry.

**Infection site.** Small intestine.

**Reported by.**Mituch (1970, 1975), Mituch et al. (1987).

##### *Catenotaenia henttoneni* Haukisalmi & Tenora, 1993

**Synonym.**Catenotaenia cricetorum f. glareolica Tenora, 1959.

**Hosts.***Apodemus flavicollis*, *Apodemus sylvaticus*, *Chionomys nivalis*, *Microtus agrestis*, *Microtus arvalis*, *Microtus subterraneus*, *Microtus tatricus*, *Mus musculus*, *Myodes glareolus*.

**Localities.** Čergov Mts., Domica, PLA Poľana, Roháčska dolina, Vysoké Tatry.

**Infection site.** Intestines, small intestine.

**Reported by.**Tenora (1963, 1967), Mituch (1970, 1975), Mészáros and Štolmann (1984), Mituch et al. (1987).

##### *Catenotaenia pusilla* (Goeze, 1782)

**Hosts.***Apodemus agrarius*, *Apodemus flavicollis*, *Apodemus sylvaticus*, *Chionomys nivalis*, *Microtus agrestis*, *Microtus arvalis*, *Microtus subterraneus*, *Microtus tatricus*, *Mus musculus*, *Myodes glareolus*, *Rattus norvegicus*.

**Localities.** Bratislava, Eastern Slovakia, Gabčíkovo, Košice, Kráľovský Chlmec, Nízke Tatry, PLA Poľana, Rožňava, South Slovakia, Vysoké Tatry.

**Infection site.** Intestines, small intestine.

**Reported by.**Erhardová (1955a, 1956, 1958), Mituch (1970, 1975).

#### ﻿Genus *Spasskijela* Tenora, 1959

##### *Spasskijela lobata* (Baer, 1925)

**Synonym.***Skrjabinotaenia lobata* (Baer, 1925).

**Hosts.***Apodemus agrarius*, *Apodemus flavicollis*, *Microtus arvalis*, *Mus musculus*, *Myodes glareolus*.

**Localities.** Bratislava, Domica, Gabčíkovo, Košice, PLA Poľana, Rozhanovce, Zádiel, Žiar nad Hronom.

**Infection site.** Intestines, small intestine.

**Reported by.**Erhardová (1956, 1958), Tenora (1963), Mituch (1975), Ondríková et al. (2010).

#### ﻿Family Dilepididae Railliet & Henry, 1909

**Genus *Sobolevitaenia* Spasskaya & Makarenko, 1965**

##### *Sobolevitaenia unicoronata* (Fuhrmann, 1908)

**Synonym.***Choanotaenia unicoronata* (Fuhrmann, 1908).

**Hosts.***Apodemus flavicollis*, *Microtus arvalis*.

**Localities.** Roháčska dolina, Vysoké Tatry.

**Infection site.** Small intestine.

**Reported by.**Tenora (1967), Mituch (1970).

**Remark.** The definitive hosts of *S. unicoronata* are passeriform birds (Spasskaya and Spasskii 1977). Nonetheless, Tenora (1967) and Mitúch (1970) each reported larvae of the species recovered from rodents. These occurrences likely represent paratenic parasitism.

#### ﻿Family Hymenolepididae Ariola, 1899

**Genus *Armadolepis* Spasskii, 1954**

##### *Armadolepis myoxi* (Rudolphi, 1819)

**Hosts.***Dryomys nitedula*, *Glis glis*.

**Localities.** PLA Poľana, Slovenský kras, Vysoké Tatry.

**Infection site.** Small intestine.

**Reported by.**Tenora (1965), Mituch (1970, 1975).

**Remark.**Tenora (1965) reported *H. sulcata* from *G. glis*. Makarikov and Georgiev (2020) re-examine the archived material of Tenora (1965) and re-identified it as *A. myoxi* (*sensu stricto*).

##### *Armadolepis spasskyi* Tenora & Baruš, 1958

**Hosts.***Dryomys nitedula*, *Eliomys quercinus*, *Glis glis*.

**Locality.** Slovenský kras.

**Infection site.** Small intestine.

**Reported by.**Tenora and Baruš (1958), Tenora (1965).

**Remark.** The species was initially described by Tenora and Baruš (1958) from *D. nitedula*, *E. quercinus*, and *G. glis*. Makarikov and Georgiev (2020) redescribed the species based on the type series, adding corrections to the diagnostic characters of the species. They also re‐examined archived cestode specimens recovered from *G. glis* in Slovakia and found no confirmed occurrences of *A. spasskyi*. Therefore, the authors concluded that the records of *A. spasskyi* from *G. glis* are doubtful.

#### ﻿Genus *Arostrilepis* Mas-Coma & Tenora, 1997

##### *Arostrilepis horrida* (Linstow, 1901)

**Hosts.***Apodemus flavicollis*, *Apodemus sylvaticus*, *Apodemus uralensis*, *Arvicola amphibius*, *Chionomys nivalis*, *Microtus agrestis*, *Microtus arvalis*, *Microtus subterraneus*, *Microtus tatricus*, *Mus musculus*, *Myodes glareolus*, *Rattus norvegicus*, *Sicista betulina*.

**Localities.** Čergov Mts., Nové Zámky, PLA Poľana, Roháčska dolina, TANAP, Vysoké Tatry.

**Infection site.** Small intestine.

**Reported by.**Mituch (1957, 1970, 1975), Tenora (1967), Mészáros and Štolmann (1984), Mituch et al. (1987).

#### ﻿Genus *Hymenolepis* Weinland, 1858

##### *Hymenolepis diminuta* (Rudolphi, 1819)

**Hosts.***Apodemus agrarius*, *Apodemus flavicollis*, *Apodemus sylvaticus*, *Apodemus uralensis*, *Eliomys quercinus*, *Glis glis*, *Microtus agrestis*, *Microtus arvalis*, *Microtus oeconomus*, *Microtus subterraneus*, *Microtus tatricus*, *Mus musculus*, *Mus spicilegus*, *Myodes glareolus*, *Rattus norvegicus*, *Spermophilus citellus*.

**Localities.** Bratislava, Čergov Mts., Domica, Gabčíkovo, Galanta, Košice, Kráľovský Chlmec, Levice, Medzilaborce, Michalovce, Nové Zámky, PLA Poľana, Prešov, Rozhanovce, Sečovce, Senec, Slovakia, Slovenský kras, Snina, South Slovakia, Šurany, Svit, Turčianske Teplice, Veľké Kapušany, Vysoké Tatry.

**Infection site.** Small intestine.

**Reported by.**Erhardová (1955a, 1955b, 1956, 1958), Mituch (1957, 1970, 1975), Tenora (1963, 1965), Mészáros and Štolmann (1984), Mituch et al. (1987), Ribas et al. (2009), Ondríková et al. (2010).

##### *Hymenolepis sulcata* (von Linstow, 1879)

**Host.***Glis glis*.

**Locality.** Rozhanovce.

**Infection site.** Small intestine.

**Reported by.**Sałamatin et al. (2005).

##### *Hymenolepis* spp.

**Hosts.***Dryomys nitedula*, *Eliomys quercinus*, *Glis glis*, *Spermophilus citellus*.

**Locality.** Bratislava – airport, Ďurkovec – camp, Chtelnica, Jánovce, Roháčska dolina, Slovenský kras, Teplička.

**Infection site.** Small intestine.

**Reported by.**Baruš and Tenora (1957), Tenora (1967), Štrkolcová et al. (2024).

**Remark.**Tenora (1967) reported a single specimen of *Hymenolepis sulcata* from *D. nitedula*. However, Makarikov and Georgiev (2020) re-examined the original archived material. They concluded that the specimen cannot be identified to the species level due to the absence of its scolex and the poor condition of the strobila. Therefore, we include this record as *Hymenolepis* sp. in the checklist.

#### ﻿Genus *Kontrimavichusia* Makarikov & Binkienė, 2022

##### *Kontrimavichusia asymmetrica* (Janicki, 1904)

**Synonyms.***Rodentolepis asymmetrica* (Janicki, 1904), *Hymenolepis ampla* Erhardová, 1955, *Rodentolepis ampla* (Erhardová, 1955).

**Hosts.***Apodemus flavicollis*, *Apodemus sylvaticus*, *Chionomys nivalis*, *Microtus agrestis*, *Microtus arvalis*, *Microtus subterraneus*, *Microtus tatricus*, *Mus musculus*, *Myodes glareolus*, *Rattus norvegicus*.

**Localities.** Bratislava, Čergov Mts., Eastern Slovakia, Gabčíkovo, Nízke Tatry, PLA Poľana, Roháčska dolina, TANAP, Vysoké Tatry.

**Infection site.** Small intestine.

**Reported by.**Erhardová (1955a, 1956, 1958), Tenora (1955, 1967), Mituch (1970, 1975), Mészáros and Štolmann (1984), Mituch et al. (1987).

#### ﻿Genus *Rodentolepis* Spasskii, 1954

##### *Rodentolepis fraterna* (Stiles, 1906)

**Hosts.***Apodemus agrarius*, *Apodemus flavicollis*, *Apodemus uralensis*, *Microtus arvalis*, *Microtus tatricus*, *Mus musculus*, *Myodes glareolus*, *Rattus norvegicus*.

**Localities.** Banská Bystrica, Dolný Kubín, Košice, Kráľovský Chlmec, Liptovský Mikuláš, Martin, Nové Mesto nad Váhom, PLA Poľana, Poprad, Púchov, Rajec, Rozhanovce, Rožňava, Ružomberok, Sečovce, Senec, Šurany, Svit, Topoľčany, Trebišov, Turčianske Teplice, Veľké Kapušany, Vysoké Tatry, Žilina, Zvolen.

**Infection site.** Small intestine.

**Reported by.**Erhardová (1955a, 1956), Mituch (1957, 1970, 1975), Ondríková et al. (2010).

##### *Rodentolepis microstoma* (Dujardin, 1845)

**Host.***Cricetus cricetus*.

**Locality.** Košice.

**Infection site.** Not specified.

**Reported by.**Jarošová et al. (2020).

##### *Rodentolepis straminea* (Goeze, 1782)

**Hosts.***Apodemus agrarius*, *Apodemus flavicollis*, *Apodemus sylvaticus*, *Microtus arvalis*, *Myodes glareolus*, *Rattus norvegicus*.

**Localities.** Domica, PLA Poľana, Rozhanovce, Vysoké Tatry.

**Infection site.** Small intestine.

**Reported by.**Tenora (1963), Mituch (1970, 1975), Mituch et al. (1987), Ondríková et al. (2010).

#### ﻿Family Mesocestoididae Fuhrman, 1907

**Genus *Mesocestoides* Vaillant, 1863**

##### *Mesocestoides* spp.

**Hosts.***Apodemus agrarius*, *Apodemus flavicollis*, *Myodes glareolus*.

**Localities.** Čergov Mts., Rozhanovce.

**Infection site.** Not specified.

**Reported by.**Mészáros and Štolmann (1984), Ondríková et al. (2010).

**Remark.***Mesocestoides* spp. are zoonotic cestodes found as adults in carnivorous domestic and wild definitive hosts and as metacestodes in several taxa of intermediate hosts, including Rodentia (Jesudoss Chelladurai and Brewer 2021).

#### ﻿Family Paruterinidae Fuhrmann, 1907

**Genus *Cladotaenia* Cohn, 1901**

##### *Cladotaenia cylindracea* (Bloch, 1782)

**Hosts.***Apodemus flavicollis*, *Myodes glareolus*.

**Localities.** PLA Poľana, TANAP, Vysoké Tatry.

**Infection site.** Liver.

**Reported by.**Mituch (1970, 1975), Mituch et al. (1987).

**Remark.** In Slovakia, species from the genus *Cladotaenia* are recorded in birds of prey (Komorová et al. 2017). Rodents are intermediate hosts for these cestodes.

#### ﻿Family Taeniidae Ludwig, 1886

**Genus *Echinococcus* Rudolphi, 1801**

##### *Echinococcus multilocularis* Leuckart, 1863

**Host.***Ondatra zibethicus*.

**Locality.** Prešov.

**Infection site.** Liver.

**Reported by.**Miterpáková et al. (2006).

**Remark.** Definitive hosts of *E. multilocularis* are canids. Rodents serve as intermediate hosts (Caira et al. 2022).

#### ﻿Genus *Hydatigera* Lamarck, 1816

##### *Hydatigera taeniaeformis* (Batsch, 1786)

**Synonym.***Strobilocercus fasciolaris*.

**Hosts.***Apodemus flavicollis*, *Apodemus sylvaticus*, *Apodemus uralensis*, *Microtus agrestis*, *Microtus arvalis*, *Microtus subterraneus*, *Microtus tatricus*, *Mus musculus*, *Myodes glareolus*, *Ondatra zibethicus*, *Rattus norvegicus*, *Sciurus vulgaris*.

**Localities.** Čergov Mts., Domica, Gabčíkovo, Galanta, Košice, Lúč na Ostrove, Nízke Tatry, PLA Poľana, Roháčska dolina, Rozhanovce, Rožňava, Slovakia, Veľké Kapušany, Vysoké Tatry.

**Infection site.** Liver, spleen.

**Reported by.**Erhardová (1955a, 1956, 1958), Tenora (1963, 1967), Mituch (1970, 1975), Mészáros and Štolmann (1984), Rajský (1985), Mituch et al. (1987), Ondríková et al. (2010).

**Remark.** A recent study demonstrated that *H. taeniaeformis* exclusively uses murine rodents (rats and mice) as intermediate hosts, with felids as definitive hosts (Lavikainen et al. 2016). Therefore, historical records from non-murine rodents should most likely be attributed to *Hydatigera kamiyai* Iwaki in Lavikainen et al. 2016.

#### ﻿Genus *Taenia* Linnaeus, 1758

##### *Taenia crassiceps* (Zeder, 1800)

**Hosts.***Apodemus flavicollis*, *Apodemus sylvaticus*, *Chionomys nivalis*, *Dryomys nitedula*, *Microtus agrestis*, *Microtus arvalis*, *Microtus oeconomus*, *Microtus subterraneus*, *Microtus tatricus*, *Mus musculus*, *Myodes glareolus*.

**Localities.** Domica, Gabčíkovo, PLA Poľana, Roháčska dolina, Vysoké Tatry.

**Infection site.** Body cavity, subcutaneous tissue.

**Reported by.**Erhardová (1955a, 1956), Tenora (1955, 1963, 1967), Mituch (1970, 1975), Mituch et al. (1987).

**Remark.***T. crassiceps* is a tapeworm described from *Vulpes vulpes* L. (Caira et al. 2022). Carnivorous mammals are definitive hosts. While rodents are intermediate hosts, they develop the metacestode (larval) stage of the parasite.

##### *Taenia martis* (Zeder, 1803)

**Host.***Myodes glareolus*.

**Locality.** Čergov Mts.

**Infection site.** Not specified.

**Reported by.**Mészáros and Štolmann (1984).

**Remark.***T. martis* is described from *Martes martes* L. (Caira et al. 2022). Rodents are intermediate hosts.

##### *Taenia pisiformis* (Bloch, 1780)

**Hosts.***Apodemus flavicollis*, *Apodemus sylvaticus*, *Mus musculus*, *Myodes glareolus*.

**Localities.** Košice, PLA Poľana, Roháčska dolina, Sklené Teplice, Vysoké Tatry.

**Infection site.** Body cavity, liver, mesentery.

**Reported by.**Erhardová (1956), Tenora (1967), Mituch (1970, 1975).

**Remark.** Definitive hosts of *T. piriformis* are canids, intermediate hosts – lagomorphs and rodents (Caira et al. 2022).

##### *Taenia polyacantha* Leuckart, 1856

**Hosts.***Apodemus flavicollis*, *Apodemus sylvaticus*, *Chionomys nivalis*, *Dryomys nitedula*, *Microtus agrestis*, *Microtus subterraneus*, *Microtus tatricus*, *Myodes glareolus*.

**Localities.** Čergov Mts., Roháčska dolina, South Slovakia, Vysoké Tatry.

**Infection site.** Abdominal cavity.

**Reported by.**Erhardová (1955a, 1956), Tenora (1967), Mituch (1970), Mészáros and Štolmann (1984), Mituch et al. (1987).

**Remark.***T. polyacantha* is described from *V. vulpes* (Caira et al. 2022). Rodents are intermediate hosts.

#### ﻿Genus *Versteria* Nakao, Lavikainen, Iwaki, Haukisalmi, Konyaev, Oku, Okamoto & Ito, 2013

##### *Versteria mustelae* (Gmelin, 1790)

**Synonyms.***Taenia tenuicollis* Rudolphi, 1819, *Taenia mustelae* Gmelin, 1790.

**Hosts.***Apodemus agrarius*, *Apodemus flavicollis*, *Chionomys nivalis*, *Microtus agrestis*, *Microtus arvalis*, *Microtus subterraneus*, *Microtus tatricus*, *Mus musculus*, *Muscardinus avellanarius*, *Myodes glareolus*, *Rattus norvegicus*.

**Localities.** Čergov Mts., Domica, Michalovce, Nízke Tatry, PLA Poľana, Prešov, Roháčska dolina, Vysoké Tatry.

**Infection site.** Liver.

**Reported by.**Erhardová (1955a, 1956, 1958), Tenora (1955, 1963, 1967), Mituch (1970, 1975), Mészáros and Štolmann (1984), Mituch et al. (1987).

**Remark.** The known definitive hosts are representatives of the genus *Martes*. Intermediate hosts are primarily rodents (Caira et al. 2022).

#### ﻿Class Trematoda Rudolphi, 1808

**Order Diplostomida Olson, Cribb, Tkach, Bray & Littlewood, 2003**

**Family Brachylaimidae Joyeux & Foley, 1930**

**Genus *Brachylaima* Dujardin, 1843**

##### *Brachylaima recurvum* Dujardin, 1845

**Hosts.***Eliomys quercinus*, *Glis glis*.

**Localities.** Jablonov, Slovenský kras.

**Infection site.** Small intestine.

**Reported by.**Baruš and Tenora (1957), Erhardová (1958).

##### *Brachylaima* spp.

**Host.***Apodemus agrarius*.

**Locality.** Rozhanovce.

**Infection site.** Small intestine.

**Reported by.**Ondríková et al. (2010).

#### ﻿Family Diplostomidae Poirier, 1886

**Genus *Alaria* Greville, 1830**

##### *Alaria alata* (Goeze, 1782)

**Host.***Apodemus flavicollis*.

**Locality.** Vysoké Tatry.

**Infection site.** Body cavity.

**Reported by.**Mituch et al. (1987).

**Remark.***A. alata* requires freshwater snails as its first intermediate host and amphibians as its second intermediate host, uses various vertebrates (e.g., rodents, wild boar) as paratenic hosts, and reaches maturity in carnivorous definitive hosts (Möhl et al. 2009).

#### ﻿Order Plagiorchiida La Rue, 1957

**Family Cladorchiidae Fischoeder, 1901**

**Genus *Neostichorchis* Jones, 2005**

##### *Neostichorchis subtriquetus* (Rudolphi, 1814)

**Host.***Microtus arvalis*.

**Locality.** Vysoké Tatry.

**Infection site.** Stomach.

**Reported by.**Mituch et al. (1987).

#### ﻿Genus *Stichorchis* Fischoeder, 1901

##### *Stichorchis subtriquetrus* (Rudolphi, 1814)

**Host.***Castor fiber*.

**Localities.** Danube River, Hanušovce nad Topľou, Laborec River, Topľa River.

**Infection site.** Caecum, colon, intestines.

**Reported by.**Bystrianska et al. (2021), Lazár et al. (2023).

#### ﻿Family Dicrocoeliidae Looss, 1899

**Genus *Dicrocoelium* Dujardin, 1845**

##### *Dicrocoelium dendriticum* (Rudolphi, 1819)

**Synonym.***Dicrocoelium lanceatum* Stiles & Hassall, 1898.

**Host.***Rattus norvegicus*.

**Locality.** Košice.

**Infection site.** Stomach.

**Reported by.**Mituch (1957).

#### ﻿Genus *Lyperosomum* Looss, 1899

##### *Lyperosomum armenicum* Shcherbakova, 1942

**Host.***Dryomys nitedula*.

**Localities.** PLA Poľana, Vysoké Tatry.

**Infection site.** Liver.

**Reported by.**Mituch (1970, 1975).

#### ﻿Family Echinostomatidae Looss, 1899

**Genus *Echinostoma* Rudolphi, 1809**

##### *Echinostoma* spp.

**Hosts.***Apodemus flavicollis*, *Myodes glareolus*.

**Locality.** Vysoké Tatry.

**Infection site.** Small intestine.

**Reported by.**Mituch (1970).

#### ﻿Family Notocotylidae Lühe, 1909

**Genus *Notocotylus* Diesing, 1839**

##### *Notocotylus noyeri* Joyeux, 1922

**Hosts.***Chionomys nivalis*, *Microtus agrestis*, *Microtus arvalis*, *Microtus oeconomus*, *Microtus subterraneus*.

**Localities.** Gabčíkovo, PLA Poľana, South Slovakia, Vysoké Tatry.

**Infection site.** Caecum, small intestine.

**Reported by.**Erhardová (1955a, 1955b, 1956), Mituch (1970, 1975), Mituch et al. (1987).

#### ﻿Genus *Quinqueserialis* Skvortsov, 1935

##### *Quinqueserialis quinqueserialis* (Barker & Laughlin, 1911)

**Host.***Microtus arvalis*.

**Locality.** PLA Poľana.

**Infection site.** Caecum.

**Reported by.**Mituch (1975).

#### ﻿Family Plagiorchiidae Lühe, 1901

**Genus *Plagiorchis* Lühe, 1899**

##### *Plagiorchis elegans* (Rudolphi, 1802)

**Hosts.***Apodemus flavicollis*, *Myodes glareolus*.

**Locality.** Vysoké Tatry.

**Infection site.** Small intestine.

**Reported by.**Mituch et al. (1987).

##### *Plagiorchis muris* Tanabe, 1922

**Hosts.***Microtus oeconomus*, *Myodes glareolus*.

**Localities.** Gabčíkovo, PLA Poľana, South Slovakia, Vysoké Tatry.

**Infection site.** Small intestine.

**Reported by.**Tenora (1955), Erhardová (1956), Mituch (1970, 1975).

##### *Plagiorchis proximus* Barker, 1915

**Host.***Apodemus flavicollis*.

**Localities.** PLA Poľana, Vysoké Tatry.

**Infection site.** Small intestine.

**Reported by.**Mituch (1970, 1975).

#### ﻿Family Psilostomidae Looss, 1900

**Genus *Psilotrema* Odhner, 1913**

##### *Psilotrema pharyngeatum* Grabda, 1954

**Host.***Microtus oeconomus*.

**Locality.** Gabčíkovo.

**Infection site.** Small intestine.

**Reported by.**Erhardová (1958).

### ﻿Host-parasite list

#### ﻿*Apodemus agrarius* (Pallas, 1771)

Nematoda – *Heligmosomoides polygyrus*, *Heterakis spumosa*, *Mastophorus muris*, Syphacia (Syphacia) obvelata, Syphacia (Syphacia) stroma, *Toxocara* spp.; Cestoda – *Catenotaenia pusilla*, *Hymenolepis diminuta*, *Mesocestoides* spp., *Paranoplocephala omphalodes*, *Rodentolepis fraterna*, *Rodentolepis straminea*, *Spasskijela lobata*, *Versteria mustelae*; Trematoda – *Brachylaima* spp.

#### ﻿*Apodemus flavicollis* (Melchior, 1834)

Acanthocephala – *Moniliformis moniliformis*; Nematoda – *Aonchotheca annulosa*, *Aspiculuris dinniki*, *Aspiculuris tetraptera*, *Calodium hepaticum*, *Heligmosomoides glareoli*, *Heligmosomoides polygyrus*, *Heligmosomum costellatum*, *Rictularia proni*, Syphacia (Seuratoxyuris) frederici, Syphacia (Syphacia) obvelata, Syphacia (Syphacia) stroma, *Toxocara* spp., *Trichinella pseudospiralis*, *Trichinella* spp., *Trichuris muris*; Cestoda – *Anoplocephaloides dentata*, *Arostrilepis horrida*, *Catenotaenia henttoneni*, *Catenotaenia pusilla*, *Cladotaenia cylindracea*, *Hydatigera taeniaeformis*, *Hymenolepis diminuta*, *Kontrimavichusia asymmetrica*, *Mesocestoides* spp., *Paranoplocephala macrocephala*, *Paranoplocephala omphalodes*, *Rodentolepis fraterna*, *Rodentolepis straminea*, *Sobolevitaenia unicoronata*, *Spasskijela lobata*, *Taenia crassiceps*, *Taenia pisiformis*, *Taenia polyacantha*, *Versteria mustelae*; Trematoda – *Alaria alata*, *Echinostoma* spp., *Plagiorchis elegans*, *Plagiorchis proximus*.

#### ﻿*Apodemus sylvaticus* (Linnaeus, 1758)

Acanthocephala – *Moniliformis moniliformis*; Nematoda – *Aonchotheca annulosa*, *Aspiculuris dinniki*, *Aspiculuris tetraptera*, *Calodium hepaticum*, *Glistrongylus gracilis*, *Heligmosomoides glareoli*, *Heligmosomoides polygyrus*, *Heligmosomum costellatum*, *Porrocaecum depressum*, *Rictularia proni*, Syphacia (Syphacia) obvelata, *Toxocara* spp., *Trichuris muris*; Cestoda – *Arostrilepis horrida*, *Catenotaenia henttoneni*, *Catenotaenia pusilla*, *Hydatigera taeniaeformis*, *Hymenolepis diminuta*, *Kontrimavichusia asymmetrica*, *Paranoplocephala macrocephala*, *Paranoplocephala omphalodes*, *Rodentolepis straminea*, *Taenia crassiceps*, *Taenia pisiformis*, *Taenia polyacantha*.

#### ﻿*Apodemus uralensis* (Pallas, 1811)

Nematoda – *Aspiculuris dinniki*, *Heligmosomoides polygyrus*, *Heligmosomum costellatum*, *Toxocara* spp.; Cestoda – *Arostrilepis horrida*, *Hydatigera taeniaeformis*, *Hymenolepis diminuta*, *Rodentolepis fraterna*.

#### ﻿*Arvicola amphibius* (Linnaeus, 1758)

Cestoda – *Arostrilepis horrida*, *Paranoplocephala macrocephala*; Nematoda – *Calodium hepaticum*.

#### ﻿*Castor fiber* Linnaeus, 1758

Nematoda – *Travassosius rufus*; Trematoda – *Stichorchis subtriquetrus*.

#### ﻿*Chionomys nivalis* (Martins, 1842)

Nematoda – *Aspiculuris dinniki*, *Calodium hepaticum*, *Heligmosomoides glareoli*, *Heligmosomum costellatum*, *Mastophorus muris*, *Pelodera orbitalis*, Syphacia (Syphacia) obvelata; Cestoda – *Anoplocephaloides dentata*, *Arostrilepis horrida*, *Catenotaenia henttoneni*, *Catenotaenia pusilla*, *Eurotaenia gracilis*, *Kontrimavichusia asymmetrica*, *Paranoplocephala macrocephala*, *Paranoplocephala omphalodes*, *Taenia crassiceps*, *Taenia polyacantha*, *Versteria mustelae*; Trematoda – *Notocotylus noyeri*.

#### ﻿*Cricetus cricetus* (Linnaeus, 1758)

Cestoda – *Rodentolepis microstoma*.

#### ﻿*Dryomys nitedula* (Pallas, 1778)

Nematoda – *Glistrongylus gracilis*, *Rictularia proni*; Cestoda – *Armadolepis myoxi*, *Armadolepis spasskyi*, *Hymenolepis* spp., *Taenia crassiceps*, *Taenia polyacantha*; Trematoda – *Lyperosomum armenicum*.

#### ﻿*Eliomys quercinus* (Linnaeus, 1766)

Nematoda – *Aonchotheca myoxinitelae*, *Rictularia proni*; Cestoda – *Armadolepis spasskyi*, *Hymenolepis diminuta*, *Hymenolepis* spp.; Trematoda – *Brachylaima recurvum*.

#### ﻿*Glis glis* (Linnaeus, 1766)

Nematoda – *Glistrongylus gracilis*; Acanthocephala – *Moniliformis moniliformis*; Cestoda – *Armadolepis myoxi*, *Armadolepis spasskyi*, *Hymenolepis diminuta*, *Hymenolepis sulcata*, *Hymenolepis* spp.; Trematoda – *Brachylaima recurvum*.

#### ﻿*Marmota marmota* (Linnaeus, 1758)

Nematoda – Syphacia (Syphacia) montana, Trichostrongylidae gen. sp.; Cestoda – *Ctenotaenia marmotae*, *Paranoplocephala* spp.

#### ﻿*Micromys minutus* (Pallas, 1771)

Nematoda – *Toxocara* spp.

#### ﻿*Microtus agrestis* (Linnaeus, 1761)

Nematoda – *Aspiculuris dinniki*, *Calodium hepaticum*, *Heligmosomoides glareoli*, *Heligmosomum costellatum*, *Heligmosomum halli*, *Pelodera orbitalis*, *Porrocaecum* spp., Syphacia (Syphacia) montana, Syphacia (Syphacia) obvelata, *Trichuris muris*; Cestoda – *Anoplocephaloides dentata*, *Arostrilepis horrida*, *Catenotaenia henttoneni*, *Catenotaenia pusilla*, *Eurotaenia gracilis*, *Hydatigera taeniaeformis*, *Hymenolepis diminuta*, *Kontrimavichusia asymmetrica*, *Paranoplocephala macrocephala*, *Paranoplocephala omphalodes*, *Taenia crassiceps*, *Taenia polyacantha*, *Versteria mustelae*; Trematoda – *Notocotylus noyeri*.

#### ﻿*Microtus arvalis* (Pallas, 1779)

Acanthocephala – *Moniliformis moniliformis*; Nematoda – *Aonchotheca annulosa*, *Calodium hepaticum*, *Carolinensis minutus*, *Glistrongylus gracilis*, *Heligmosomoides polygyrus*, *Heligmosomum costellatum*, *Heligmosomum halli*, *Pelodera orbitalis*, *Rictularia proni*, Syphacia (Syphacia) nigeriana, Syphacia (Syphacia) obvelata, *Toxocara* spp., *Trichostrongylus retortaeformis*, *Trichuris muris*; Cestoda – *Anoplocephaloides dentata*, *Arostrilepis horrida*, *Catenotaenia henttoneni*, *Catenotaenia pusilla*, *Hydatigera taeniaeformis*, *Hymenolepis diminuta*, *Kontrimavichusia asymmetrica*, *Paranoplocephala macrocephala*, *Paranoplocephala microti*, *Paranoplocephala omphalodes*, *Rodentolepis fraterna*, *Rodentolepis straminea*, *Sobolevitaenia unicoronata*, *Spasskijela lobata*, *Taenia crassiceps*, *Versteria mustelae*; Trematoda – *Neostichorchis subtriquetus*, *Notocotylus noyeri*, *Quinqueserialis quinqueserialis*.

#### ﻿*Microtus oeconomus* (Pallas, 1776)

Nematoda – *Heligmosomoides polygyrus*, Syphacia (Syphacia) obvelata, *Trichuris muris*; Cestoda – *Hymenolepis diminuta*, *Paranoplocephala macrocephala*, *Taenia crassiceps*; Trematoda – *Notocotylus noyeri*, *Plagiorchis muris*, *Psilotrema pharyngeatum*.

#### ﻿*Microtus subterraneus* (Selys-Longchamps, 1836)

Nematoda – *Aspiculuris dinniki*, *Carolinensis minutus*, *Heligmosomoides glareoli*, *Heligmosomoides polygyrus*, *Heligmosomum costellatum*, *Heligmosomum halli*, *Mastophorus muris*, *Pelodera orbitalis*, *Porrocaecum* spp., Syphacia (Syphacia) montana, Syphacia (Syphacia) obvelata; Cestoda – *Anoplocephaloides dentata*, *Arostrilepis horrida*, *Catenotaenia henttoneni*, *Catenotaenia pusilla*, *Eurotaenia gracilis*, *Hydatigera taeniaeformis*, *Hymenolepis diminuta*, *Kontrimavichusia asymmetrica*, *Microticola blanchardi*, *Paranoplocephala macrocephala*, *Paranoplocephala omphalodes*, *Paranoplocephala* spp., *Taenia crassiceps*, *Taenia polyacantha*, *Versteria mustelae*; Trematoda – *Notocotylus noyeri*.

#### ﻿*Microtus tatricus* Kratochvíl, 1952

Nematoda – *Aspiculuris dinniki*, *Heligmosomoides glareoli*, *Heligmosomum costellatum*, *Heligmosomum halli*, *Pelodera orbitalis*, Syphacia (Syphacia) montana; Cestoda – *Anoplocephaloides dentata*, *Arostrilepis horrida*, *Catenotaenia henttoneni*, *Catenotaenia pusilla*, *Eurotaenia gracilis*, *Hydatigera taeniaeformis*, *Hymenolepis diminuta*, *Kontrimavichusia asymmetrica*, *Microticola blanchardi*, *Paranoplocephala macrocephala*, *Paranoplocephala omphalodes*, *Rodentolepis fraterna*, *Taenia crassiceps*, *Taenia polyacantha*, *Versteria mustelae*.

#### ﻿*Mus musculus* Linnaeus, 1758

Acanthocephala – *Moniliformis moniliformis*; Cestoda – *Arostrilepis horrida*, *Catenotaenia henttoneni*, *Catenotaenia pusilla*, *Hydatigera taeniaeformis*, *Hymenolepis diminuta*, *Kontrimavichusia asymmetrica*, *Paranoplocephala macrocephala*, *Rodentolepis fraterna*, *Spasskijela lobata*, *Taenia crassiceps*, *Taenia pisiformis*, *Versteria mustelae*; Nematoda – *Aonchotheca annulosa*, *Aspiculuris tetraptera*, *Heligmosomoides polygyrus*, *Heligmosomum costellatum*, *Mastophorus muris*, *Porrocaecum depressum*, *Rictularia proni*, Syphacia (Syphacia) obvelata, *Toxocara* spp., *Trichuris muris*.

#### ﻿*Mus spicilegus* Petényi, 1882

Nematoda – *Heligmosomoides polygyrus*, Syphacia (Syphacia) obvelata; Cestoda – *Hymenolepis diminuta*.

#### ﻿*Muscardinus avellanarius* (Linnaeus, 1758)

Nematoda – *Heligmosomoides polygyrus*, Syphacia (Syphacia) obvelata; Cestoda – *Versteria mustelae*.

#### ﻿*Myodes glareolus* (Schreber, 1780)

Acanthocephala – *Moniliformis moniliformis*; Nematoda – *Aonchotheca annulosa*, *Aspiculuris dinniki*, *Calodium hepaticum*, *Carolinensis minutus*, *Heligmosomoides glareoli*, *Heligmosomoides polygyrus*, *Heligmosomum costellatum*, *Heligmosomum halli*, *Heligmosomum mixtum*, *Mastophorus muris*, *Pelodera orbitalis*, *Rictularia proni*, *Rictularia* spp., Syphacia (Syphacia) montana, Syphacia (Syphacia) obvelata, *Toxocara* spp.; Cestoda – *Anoplocephaloides dentata*, *Arostrilepis horrida*, *Catenotaenia henttoneni*, *Catenotaenia pusilla*, *Cladotaenia cylindracea*, *Eurotaenia gracilis*, *Hydatigera taeniaeformis*, *Hymenolepis diminuta*, *Kontrimavichusia asymmetrica*, *Mesocestoides* spp., *Microticola blanchardi*, *Paranoplocephala macrocephala*, *Paranoplocephala microti*, *Paranoplocephala omphalodes*, *Paranoplocephala* spp., *Rodentolepis fraterna*, *Rodentolepis straminea*, *Spasskijela lobata*, *Taenia crassiceps*, *Taenia martis*, *Taenia pisiformis*, *Taenia polyacantha*, *Versteria mustelae*; Trematoda – *Echinostoma* spp., *Plagiorchis elegans*, *Plagiorchis muris*.

#### ﻿*Ondatra zibethicus* (Linnaeus, 1766)

Cestoda – *Echinococcus multilocularis*, *Hydatigera taeniaeformis*.

#### ﻿*Rattus norvegicus* (Berkenhout, 1769)

Acanthocephala – *Moniliformis moniliformis*; Nematoda – *Aonchotheca annulosa*, *Calodium hepaticum*, *Heterakis spumosa*, *Liniscus papillosus*, *Mastophorus muris*, *Strongyloides ratti*, *Trichinella pseudospiralis*, *Trichinella* spp., *Trichosomoides crassicauda*, *Trichuris muris*; Cestoda – *Arostrilepis horrida*, *Catenotaenia pusilla*, *Hydatigera taeniaeformis*, *Hymenolepis diminuta*, *Kontrimavichusia asymmetrica*, *Paranoplocephala omphalodes*, *Rodentolepis fraterna*, *Rodentolepis straminea*, *Versteria mustelae*; Trematoda – *Dicrocoelium dendriticum*.

#### ﻿*Sciurus vulgaris* Linnaeus, 1758

Nematoda – Enterobius (Enterobius) sciuri, *Porrocaecum depressum*, *Trichostrongylus retortaeformis*; Cestoda – *Catenotaenia dendritica*, *Hydatigera taeniaeformis*.

#### ﻿*Sicista betulina* (Pallas, 1779)

Nematoda – *Heligmosomoides polygyrus*; Cestoda – *Arostrilepis horrida*.

#### ﻿*Spermophilus citellus* (Linnaeus, 1766)

Acanthocephala – *Moniliformis moniliformis*; Cestoda – *Hymenolepis diminuta*, Hymenolepis spp.; Nematoda – *Capillaria* spp., *Citellina* spp., Trichostrongylidae gen. sp.

## ﻿Discussion

The presented checklist summarises information on the helminth fauna of rodents inhabiting Slovakia. Although the list includes extensive data on helminth species from 27 rodent species, encompassing the entire available research period and information from across the country, its completeness may be limited for several reasons. These include incomplete taxonomic coverage of hosts, uneven spatial and temporal coverage of research, and methodological changes in studies.

Thirty-one rodent species have been reported in Slovakia, but two of them, *Sicista subtilis* (Pallas, 1773) and *Rattus rattus* (Linnaeus, 1758), are currently unconfirmed in the country (Krištofík and Danko 2012). Thus, of the 29 rodent species presently found in Slovakia, data on their helminths are available for 27 of them. Information on the helminths of two rodent species – *Myocastor coypus* (Molina, 1782) and *Arvicola scherman* (Shaw, 1801) – is absent, which is related to the limited distribution of these species in the country. In particular, *M. coypus* is an introduced species from South America with a fragmented distribution in Slovakia, and *A. scherman* is a rare species with only a single documented occurrence (Krištofík and Danko 2012).

Although data on helminths of rodents were collected across Slovakia, the intensity of research varied significantly between regions. The highest number of studies was conducted in Eastern Slovakia, with the Tatra National Park being the most extensively studied area, particularly in the 1950s and 1980s (see Suppl. material 1). Furthermore, research effort was unevenly distributed across host species, with most rodent species being extensively studied, while the helminth diversity of others remains poorly documented.

Different methods were used for helminth identification in the studies underlying this checklist, including microscopic examination of morphological features, coprological analysis, enzyme-linked immunosorbent assay (ELISA), and polymerase chain reaction (PCR). However, in most of these studies, morphological identification was the only approach used. Although morphology is fundamental to the taxonomy and systematics of helminths, morphological identification has certain limitations (Miller et al. 2024). This limitation is especially acute for cryptic and closely related species, which often remain unrecognised or misidentified when morphological features are the only criteria used. Molecular techniques have greatly expanded diagnostic capabilities; nonetheless, morphological examination often remains the most economical and rapid means of identifying well-characterised helminths.

Our comparison with the most comprehensive Polish helminth catalogue (Pojmańska et al. 2007) revealed 71 helminth species recorded in Poland from 17 rodent hosts: 23 cestodes, 11 trematodes, 36 nematodes, and one acanthocephalan. Although the overall helminth species richness is similar to that of Slovakia, host coverage differs. It is worth noting that Slovakia and Poland share an almost identical rodent fauna (Krištofík and Danko 2012; Tykarski 2022); *M. spicilegus* occurs only in Slovakia, whereas *Spermophilus suslicus* (Güldenstaedt, 1770) is found exclusively in Poland. In Slovakia, helminthological data are available for 27 of the 31 rodent species known to occur in the country, whereas in Poland, records exist for only 17 of the 31 species. Subsequent comparison reveals 44 helminth species shared by both countries, 29 unique to Slovakia, and 27 unique to Poland, resulting in a Jaccard similarity of 44%. Although our comparison is crude, likely influenced by biases discussed above, it suggests a high probability of finding additional helminths of rodents in Slovakia, particularly in border areas.

Since Krištofík and Danko (2012), five additional helminths (*R. microstoma*, *S. subtriquetrus*, *Citellina* spp., *T. britovi*, and *T. rufus*) have been recorded in Slovakia, highlighting the potential for further discoveries. We expect that helminth species new to Slovakia will most likely be found in their larval stages. Currently, of the 77 helminth taxa documented in Slovak rodents, 13 are documented at larval stages (*Porrocaecum* spp., *Toxocara* spp., *C. cylindracea*, all representatives of the family Taeniidae, *Mesocestoides* spp., and *A. alata*), and one appears to be a case of accidental infection (*T. retortaeformis*).

As previously mentioned, this checklist is partly based on reports produced at the Institute of Parasitology, Slovak Academy of Sciences (Mituch 1956, 1957, 1970, 1975; Mituch et al. 1987). Voucher material generated during those studies was deposited in the Institute’s collection. In 1996, the entire collection, including numerous helminths from hosts other than rodents, was transferred to the East-Slovak Museum (Košice, Slovakia), where it is still curated. The most valuable specimens, notably type material, have since been catalogued by Dudiňák and Špakulová (2005). Although the present review relies solely on literature sources, we mention the collection here for the benefit of future researchers interested in examining the original material.

In conclusion, although the present checklist provides a comprehensive synthesis of available data on rodent helminths in Slovakia, our knowledge of their diversity remains incomplete. Further research involving broader host sampling, geographically balanced study designs, and modern molecular techniques for parasite identification is needed to advance understanding in Slovakia.

## ﻿Additional information

### Use of AI

During the preparation of this article, the authors used Grammarly (https://www.grammarly.com/) to correct grammatical errors and enhance readability. After using this tool, the authors reviewed and edited the content as needed. The authors accept full responsibility for the content of the published article.

### Author ORCIDs

Yaroslav Syrota https://orcid.org/0000-0002-8070-9823

Zuzana Hurníková https://orcid.org/0000-0002-6816-2992

Vitaliy Kharchenko https://orcid.org/0000-0002-3824-2078

Martina Miterpáková https://orcid.org/0000-0002-3742-948X

### Data availability

All of the data that support the findings of this study are available in the main text or Supplementary Information.

## Supplementary materials

Supplementary material 1 Detailed information for each locality Data type (measurement/occurrence/multimedia/etc.) Explanation note This file is an Excel workbook. It consists of three sheets: data, locations, and references. The data sheet systematically compiles the information presented in the main text of the article into a tabular format optimised for computational processing. The locations sheet contains geographic information, including approximate coordinates, for each locality mentioned in the data sheet. The references sheet lists the bibliographic sources used in compiling the dataset. https://binary.pensoft.net/file/1408522This dataset is made available under the Open Database License (http://opendatacommons.org/licenses/odbl/1.0/). The Open Database License (ODbL) is a license agreement intended to allow users to freely share, modify, and use this Dataset while maintaining this same freedom for others, provided that the original source and author(s) are credited.Yaroslav Syrota, Zuzana Hurníková, Vitaliy Kharchenko, Martina Miterpáková
